# Cross-Species BAC Mapping Highlights Conservation of Chromosome Synteny across Dragon Lizards (Squamata: Agamidae)

**DOI:** 10.3390/genes11060698

**Published:** 2020-06-25

**Authors:** Shayer Mahmood Ibney Alam, Marie Altmanová, Tulyawat Prasongmaneerut, Arthur Georges, Stephen D. Sarre, Stuart V. Nielsen, Tony Gamble, Kornsorn Srikulnath, Michail Rovatsos, Lukáš Kratochvíl, Tariq Ezaz

**Affiliations:** 1 Institute for Applied Ecology, University of Canberra, Bruce, ACT 2617, Australia; georges@aerg.canberra.edu.au (A.G.); stephen.sarre@canberra.edu.au (S.D.S.); 2 Department of Ecology, Faculty of Science, Charles University, 12844 Prague, Czech Republic; marie.altmanova@natur.cuni.cz (M.A.); michail.rovatsos@natur.cuni.cz (M.R.); lukas.kratochvil@natur.cuni.cz (L.K.); 3 Laboratory of Animal Cytogenetics & Comparative Genomics, Department of Genetics, Faculty of Science, Kasetsart University, Bangkok 10900, Thailand; tulyawat.pr@ku.th (T.P.); kornsorn.s@ku.ac.th (K.S.); 4 Department of Biological Sciences, Marquette University, Milwaukee, WI 53233, USA; stunie@gmail.com (S.V.N.); anthony.gamble@marquette.edu (T.G.); 5 Division of Herpetology, Florida Museum of Natural History, University of Florida, Gainesville, FL 32611, USA; 6 Milwaukee Public Museum, 800 W. Wells St., Milwaukee, WI 53233, USA; 7 Bell Museum of Natural History, University of Minnesota, 2088 Larpenteur Ave. W., St. Paul, MN 55113, USA

**Keywords:** agamid lizards, sex chromosomes, BACs, synteny, evolution, FISH

## Abstract

Dragon lizards (Squamata: Agamidae) comprise about 520 species in six subfamilies distributed across Asia, Australasia and Africa. Only five species are known to have sex chromosomes. All of them possess ZZ/ZW sex chromosomes, which are microchromosomes in four species from the subfamily Amphibolurinae, but much larger in *Phrynocephalus vlangalii* from the subfamily Agaminae. In most previous studies of these sex chromosomes, the focus has been on Australian species from the subfamily Amphibolurinae, but only the sex chromosomes of the Australian central bearded dragon (*Pogona vitticeps*) are well-characterized cytogenetically. To determine the level of synteny of the sex chromosomes of *P. vitticeps* across agamid subfamilies, we performed cross-species two-colour FISH using two bacterial artificial chromosome (BAC) clones from the pseudo-autosomal regions of *P. vitticeps*. We mapped these two BACs across representative species from all six subfamilies as well as two species of chameleons, the sister group to agamids. We found that one of these BAC sequences is conserved in macrochromosomes and the other in microchromosomes across the agamid lineages. However, within the Amphibolurinae, there is evidence of multiple chromosomal rearrangements with one of the BACs mapping to the second-largest chromosome pair and to the microchromosomes in multiple species including the sex chromosomes of *P. vitticeps*. Intriguingly, no hybridization signal was observed in chameleons for either of these BACs, suggesting a likely agamid origin of these sequences. Our study shows lineage-specific evolution of sequences/syntenic blocks and successive rearrangements and reveals a complex history of sequences leading to their association with important biological processes such as the evolution of sex chromosomes and sex determination.

## 1. Introduction

Reptiles are well known for their diverse modes of sex determination and sex chromosomes [1,2]. They exhibit large variability in the degree of differentiation of sex chromosomes ranging from homomorphic to heteromorphic in structure [2,3,4,5]. Squamate reptiles (lizards, snakes and amphisbaenians) are the most diverse reptile group in terms of species diversity and mode of sex determination [1,6]. The variability seen among squamate sex chromosomes suggests that sex chromosome and sex determination systems have evolved independently many times. Non-homologous sex chromosomes have been reported even among relatively closely related species [2,5,7]. The same parts of the genome (i.e., homologous regions) have been found to play the role of sex chromosomes in different vertebrate taxa [5,8,9,10,11]. A high degree of synteny has been observed between birds and squamate reptiles owing to a relative low degree of chromosomal rearrangements in this group [5,12,13,14,15,16,17,18]. Temperature-dependent sex determination (TSD), genotypic sex determination (GSD), and GSD with temperature influences between relatively closely related species make squamate lizards an interesting group to study and understand the evolution of sex chromosomes.

**Figure 1:**
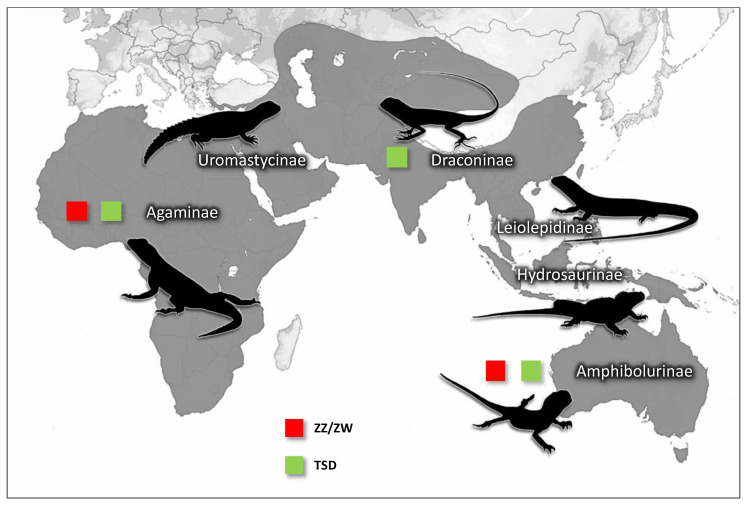
Estimated distribution of the agamid subfamilies together with known sex determination mechanisms [6,17,20,21,24,31,34,35,36,37,38,39,40,41,42,43,44,45,46,47,48,49]. The species of the subfamily Draconinae are distributed over South and Southeast Asia, Agaminae across Africa and Asia, Amphibolurinae across Australia, Papua New Guinea and Southeast Asia, Hydrosaurinae across Papua New Guinea, the Philippines and Indonesia, Leiolepidinae across Southeast Asia and Uromastycinae across Africa and South Asia. TSD—temperature dependent sex determination, ZZ/ZW—female heterogamety. Obligatory parthenogenesis has been reported in several species of the subfamily Leiolepidinae, although the sex determination system is not known in this lineage.

Agamid lizards (Squamata: Agamidae), commonly known as dragons in Australasia, are notorious for their variability in forms of sex determination [19,20,21,22,23]. Together with chameleons (Chamaeleonidae), they form the iguanian clade Acrodonta, sister to iguanas (Pleurodonta) [24,25]. Acrodonts are an interesting group in terms of the evolution and diversity of sex determination [7,26,27], while the iguanas have, with one exception (basilisks), conserved XX/XY sex chromosomes [28,29]. There are about 520 currently described agamid species [6] comprising six subfamilies that diverged around 70–120 million years ago [25,30]. Most agamid species are oviparous [6], and the groups includes species with obligate and facultative parthenogenesis [31,32,33]. Sex determination mode is relatively well studied in a few species from the subfamily Amphibolurinae [7,19], but not in the other five subfamilies (Figure 1), highlighting a significant gap in our understanding of how sex chromosomes evolved in this widespread and chromosomally variable family.

Only about one fifth (91 species) of Agamid species have been karyotyped, with diploid chromosome numbers ranging from 2n = 32 to 2n = 54 [4,6,50]. Agamids exhibit a diverse array of sex-determination mechanisms that include TSD, GSD and GSD with sex reversal [1,2,22,39,51]. Sex chromosomes have only been identified in five species, all with a female heterogametic system (ZZ male/ZW female). Sex chromosomes in an Asian species *Phrynocephalus vlangalii* from the subfamily Agaminae are macrochromosomes [39], whereas the four Australian species from the subfamily Amphibolurinae, namely, *Pogona vitticeps, P. barbata, Diporiphora nobbi* and *Ctenophorus fordi,* have micro sex chromosomes [7,20]. The karyotypes of the Australian species are highly conserved, comprising six pairs of macrochromosomes and ten pairs of microchromosomes [35]. Nevertheless, they show considerable evolutionary lability in sex determination mechanisms [19,52] with a number of likely transitions reported within GSD forms and between GSD and TSD [2,7,30,53].

Molecular cytogenetics is a powerful tool for discovering homology and evolutionary trends in reptile sex chromosomes [54,55] and has provided evidence that the sex chromosomes of lizards are extremely varied in terms of morphology and homology [2,5]. The Australian central bearded dragon, *Pogona vitticeps*, has a well-annotated genome with well-characterized ZZ/ZW sex microchromosomes, homologous to chicken chromosomes 17 and 23 [20,56,57,58,59,60]. Comparative studies based on this and other species have revealed chromosomal rearrangements involving sex chromosomes and transitions in sex chromosomes within the Amphibolurinae [7,61,62], including the rapid evolution of non-homologous ZW sex chromosomes. Here, we evaluate the synteny of sex chromosomes across the dragons of the family Agamidae using fluorescence in situ hybridization (FISH) [7]. We used two BAC clones (Pv03_L07 and Pv150_H19) derived from *P. vitticeps* ZW sex chromosomes as probes and hybridized them to the metaphase chromosomes of 14 acrodont taxa (12 agamids from all six subfamilies, and two chameleons), comprising species that span the spectrum of sex determination, including TSD, GSD and obligatory parthenogenesis.

## 2. Materials and Methods

### 2.1. Animal and Sample Collection 

In total, 22 individuals of 14 species of acrodont lizards—12 agamids (from six subfamilies) and 2 chamaeleonid species—were chosen for the study (Table 1). Animal collection, handling, sampling and all other relevant procedures for the Australian species (*P. vitticeps*, *Tympanocryptis lineata* and *Rankinia diemensis*) were performed following the Animal Ethics Guidelines of the University of Canberra (approval number CEAE 16-21), with permits issued by the ACT Government (license number LT2017960). Fieldwork conducted for *Agama picticauda* was under Miami-Dade County Parks and Recreation Scientific Research Permit number 263-2016 and Marquette University IACUC AR-288. *Calotes versicolor* and *Leiolepis reevesii rubritaeniata* specimen collection, animal care and procedures were approved by the Animal Experiment Committee, Kasetsart University, Thailand (approval number ACKU61-SCI-021). *Phrynocephalus* cf. *guttatus*, *Bronchocela cristatella*, *Leiolepis* cf. *ngovantrii*, *Saara loricata* and *Chamaeleo calyptratus* were sampled in collaboration with breeders in Czech Republic. Samples of *Hydrosaurus weberi* and *Trioceros johnstoni* were provided by Czech zoological gardens (Zoo Plzeň and Zoopark Zájezd, respectively). All experimental procedures in Czech Republic were approved by the Committee for Animal Welfare of the Ministry of Agriculture of the Czech Republic, permissions No. 29555/2006-30 and 8604/2019-7.

### 2.2. Cell Culture and Chromosome Preparation 

Fibroblast cells were cultured from the tail tissues of *P. vitticeps*, *T. lineata*, *R. diemensis* and *A. picticauda* for the cytogenetic analyses. Cells were cultured, and metaphase chromosomes were harvested following the procedures as described by Ezaz et al. [63]. *C. versicolor* and *L. reevesii rubritaeniata* cells were also cultured from tail tissues. Cell culture and chromosome harvesting followed the procedures as described by Chaiprasertsri et al. [64]. Mitotic chromosomes of *P.* cf. *guttatus*, *B. cristatella*, *L.* cf. *ngovantrii*, *S. loricata*, *C. calyptratus*, *H. weberi* and *T. johnstoni* were obtained by cultivation of leukocytes and the preparation of the cell cultures and chromosome harvesting followed a detailed protocol described in Mazzoleni et al. [65].

### 2.3. Fluorescence In Situ Hybridization (FISH) and Image Analysis

Two *P. vitticeps* ZW sex chromosome BAC clones (Pv03_L07 and Pv150_H19) from the *P. vitticeps* Bacterial Artificial Chromosome (BAC) library (6.2x, Amplicon Express, Pullman, WA, USA) [56] were mapped onto the metaphase chromosomes of all 14 species (Table 1). The sex chromosomes of *P. vitticeps* have been found to be highly repetitive in nature [56]. The BAC Pv03_L07 (about 98 kb) contains 41% of repetitive sequences of which 43% are non-LTR (long terminal repeat) retrotransposons and includes at least two genes, *ZNF135*-*like* and a fragment of *ORPRD1* [56]. BAC Pv150_H19 (size not estimated and repeat content not known) contains the *NR5A1* gene [59], which is known to play an important role in sex differentiation [59]. These two BAC clones share homologous sequences with chicken chromosome 17 and were chosen because they were previously mapped in few agamid species, and their sequence content is known [59]. The two BACs, Pv03_L07 and Pv150_H19, represent the two ends of Z and W chromosomes of *P. vitticeps*. In addition, BAC Pv03_L07 hybridizes onto the telomeric region of the second-largest chromosome (chromosome 2) of *P. vitticeps* [56,57,59]. The two BAC clones were mapped using FISH, following the protocols described in Ezaz et al. [7] and Young et al. [58].

All slides were observed, and images of metaphases were captured using a Zeiss Axio Scope A1 epifluorescence microscope fitted with a high-resolution microscopy camera AxioCam MRm Rev. 3 (Carl Zeiss Ltd. Oberkochen, Germany). Images were analyzed using Metasystems Isis FISH Imaging System V 5.5.10 software (Metasystems, Altlussheim, Germany). 

## 3. Results

**Figure 2:**
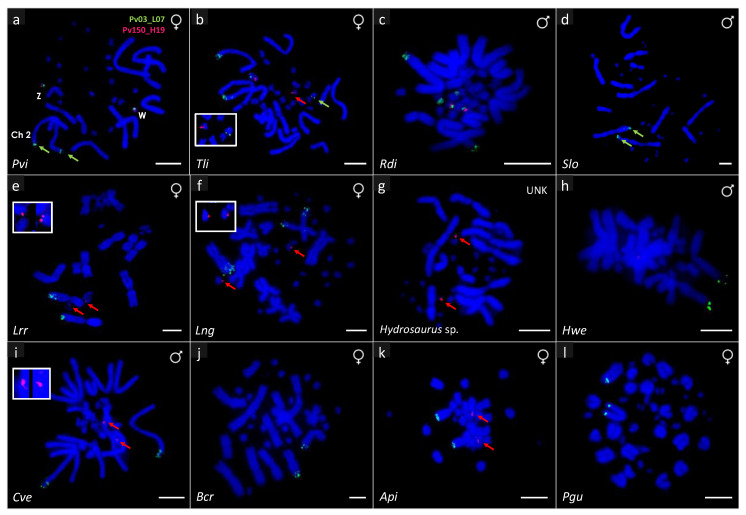
FISH (fluorescence in situ hybridization) using *P. vitticeps* BAC clones (Pv03_L07 in green and Pv150_H19 in red) on different agamid species. Pvi—*P. vitticeps* (**a**); Tli—*T. lineata* (**b**); Rdi—*R. diemensis* (**c**); Slo—*S. loricate* (**d**); Api—*A. picticauda* (**k**); Pgu—*P*. cf. *guttatus* (**l**); Cve—*C. versicolor* (**i**); Bcr—*B. cristatella* (**j**); Lrr—*L. reevesii rubritaeniata* (**e**); Lng—*L.* cf. *ngovantrii* (**f**); *Hydrosaurus* sp. (**g**); Hwe—*H. weberi* (**h**); UNK—unknown sex. Arrows and insets showing very low hybridization signals. Scale bars equal 5 µm.

In line with the previous observations [56,59,61], the BAC clone Pv03_L07 hybridized onto the Z and W chromosomes as well as onto the telomeric region of the long arms of the chromosome pair 2 in *P. vitticeps* (Figure 2a). This BAC probe hybridized onto the telomeric region of the long arms of the chromosome pair 2 in all species under the subfamilies Amphibolurinae (*P. vitticeps*, *T. lineata* and *R. diemensis*; Figure 2a–c), Uromastycinae (*S. loricata*; Figure 2d) and Leiolepidinae (*L. reevesii rubritaeniata* and *L.* cf. *ngovantrii*; Figure 2e,f). A similar hybridization pattern was also observed in *H. weberi* (Hydrosaurinae, Figure 2h), but no hybridization signal was detected in *Hydrosaurus* sp. (Figure 2g). The probe also hybridized onto chromosome 2 in *C. versicolor* from the subfamily Draconinae, (Figure 2i) but onto the fifth-largest chromosome pair in another member of that family, *B. cristatella* (Figure 2j). Hybridization signals from BAC clone Pv03_L07 were observed in the largest chromosome pair in members of the subfamily Agaminae (*A. picticauda* and *P.* cf. *guttatus*; Figure 2k,l). Additional to chromosome 2, BAC Pv03_L07 only hybridized onto microchromosomes in the subfamily Amphibolurinae (Figure 2a–c), onto one pair in *P. vitticeps* and *T. lineata* and two pairs in *R. diemensis*. In *P. vitticeps*, the BAC Pv03_L07 hybridization signal varied between Z and W with a brighter signal in the W [56,59]. The only other species in which we observed a similar pattern was *R. diemensis*. In this species, BAC Pv03_L07 hybridized to an additional pair of microchromosomes and the hybridization signals in one pair are brighter than the other. However, no inter-sex pattern variation was observed either in this species.

The hybridization patterns formed by the BAC probe Pv150_H19 across agamid lizards are presented in Figure 2. This BAC probe hybridized onto the Z and W chromosomes of *P. vitticeps* (Figure 2a), as previously observed [57,59]. The hybridization signals from Pv150_H19 co-localized with the signals from Pv03_L07 in this species (Figure 2a) and hybridized onto a pair of microchromosomes in all species of the subfamilies Amphibolurinae (*P. vitticeps*, *T. lineata* and *R. diemensis*; Figure 2a–c) and Leiolepidinae (*L. reevesii rubritaeniata* and *L*. cf. *ngovantrii*; Figure 2e,f). However, the hybridization signals were observed in only one species from each of the subfamilies Hydrosaurinae (*Hydrosaurus* sp.; Figure 2g), Draconinae (*C. versicolor*; Figure 2i) and Agaminae (*A. picticauda*; Figure 2k) while no hybridization signal was observed in *S. loricata* (subfamily Uromastycinae; Figure 2d). Both BAC clones hybridized onto microchromosomes in all species of the subfamily Amphibolurinae (*P. vitticeps, T. lineata* and *R. diemensis*; Figure 2a–c). Nevertheless, in *T. lineata*, the BACs did not colocalize on the same pair of microchromosomes. No inter- or intra-sex variation of the BAC Pv150_H19 hybridization signal was recorded from *P. vitticeps* [59], nor was variation detected in any of our studied species. No hybridization signal was observed from any of the BAC clones in any of the chameleon species (*C. calyptratus* and *T. johnstoni*; Table 1). A summary of the overall BAC mapping is presented in Table 1.

## 4. Discussion

**Figure 3:**
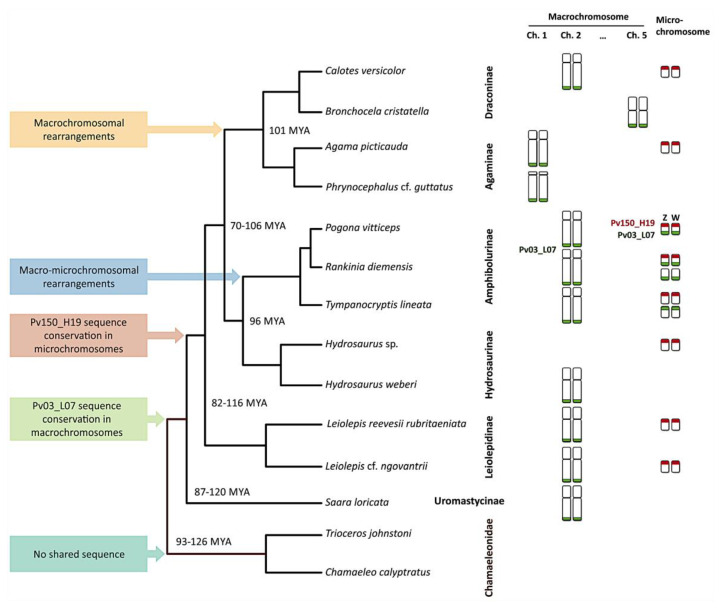
Cross-species chromosome mapping of *P. vitticeps* sex-chromosome-derived BAC probes Pv03_L07 (in green) and Pv150_H19 (in red) highlighting hypothetical evolutionary scenarios of chromosome rearrangements within the subfamilies of Agamidae. Truncated phylogeny (not according to scale) is adopted from Pyron, et al. [24]. Known divergence times are provided in million years ago (MYA) [25,30,66].

Our data revealed the conservation of macro- and microchromosome specific sequences across Agamidae. The *P. vitticeps* sex chromosome derived BAC probe Pv03_L07, which hybridizes onto the sex microchromosomes and telomeric region of chromosome 2 in *P. vitticeps*, hybridized to a pair of macrochromosomes across agamid lineages in all but one species (*Hydrosaurus* sp.; Figure 2g). This suggests that chromosomal synteny is retained across agamid lineages. In contrast, none of the BACs hybridized to chameleon chromosomes. Together, our findings indicate that the sequence is conserved in macrochromosomes across the Agamidae but has most likely been secondarily lost in the ancestor of *Hydrosaurus* sp. (Figure 3).

The BAC Pv03_L07 exhibits a conserved hybridization pattern on the telomeric region of macrochromosome 2 in members of the subfamilies Amphibolurinae, Hydrosaurinae, Leiolepidinae and Uromastycinae. However, it is localized in the largest chromosome pair in both members of the subfamily Agaminae, which might represent a synapomorphy. The localization of the hybridization signal of Pv03_L07 on chromosome 5 in *B. cristatella* suggests a chromosomal rearrangement in its ancestor. Additionally, it hybridizes onto two pairs of microchromosomes in *R. diemensis* and one pair in *T. lineata*. Both of those species are representatives of the subfamily Amphibolurinae, and so, these data lend support to the chromosomal rearrangements such as duplication near the telomeric region of ancestral chromosome 2 and successive translocation to microchromosomes as previously reported by Matsubara et al. [61].

The second BAC clone, Pv150_H19, was derived from *P. vitticeps* Z and W sex chromosomes only, and its sequences are located onto the opposite ends of the Z and W micro sex chromosomes in relation to Pv03_L07 (Figure 2a). This probe also showed somewhat conserved distribution in a pair of microchromosomes across the agamid phylogeny. The probe hybridized to all species of Amhibolurinae and Leiolepidinae, to one of two species in Hydrosaurinae, Draconinae and Agaminae and did not hybridize to the only species from Uromastycinae (Figure 3). This suggests a haphazard distribution across the lineages. The absence of signal in *S. loricata* and presence in all other agamid subfamilies indicate that BAC Pv150_H19 sequence might have evolved after the split of the other lineages from Uromastycinae (Figure 3). The lack of hybridization signal in *B. cristatella*, *P.* cf. *guttatus* and *H. weberi* suggests an independent loss in these three species. Alternatively, there could be a mutation in the target sequence so that the probe was washed away from the less complementary target, and/or shrinkage of the target sequence, so it was no longer detectable. Since BACs are usually predominately composed of repeats which evolve quickly, it is possible that the sequences are still present in all the species but no longer detectable with the approach used. The sequence content of both BACs is enriched on repetitive elements [52,55], which—due to their fast-evolution nature—may have diverged significantly since agamid and chameleon lineages split approximately 90–125 million years ago (MYA) [25,26,62]. Therefore, the homologous sequences might exist in the genome of chameleons but the BACs could not hybridize because of significant divergence from *P. vitticeps*. (These results must be viewed with some caution, however, as they are based on a limited number of chameleon species, which have also been shown to harbour transitions between sex determining systems [27].) Pv150_H19 hybridized to a microchromosome pair in both TSD and GSD species, but we were unable to determine whether these microchromosomes (with Pv150_H19 signals) were sex chromosomes. Nevertheless, since this BAC contains a gene associated with sex differentiation function (*NR5A1*), it is possible that the microchromosome pair with Pv150_H19 could be a sex chromosome in the GSD species. If so, those same homologous chromosomes could be autosomes in the TSD species while still contributing to the sex-differentiation cascade or pathways. Further investigation is required on this aspect.

The ancestral vertebrate karyotype has remained relatively stable over the last ~370 million years as large segments of ancestral chromosomes are still retained among all lineages [67]. These segments have been rearranged, but their synteny has been maintained together with increases and decreases of genomic content and genome sizes. Chromosome painting has been used to determine such homologies, as well as rearrangements among and between different reptilian species [8]. For example, karyotype and genome organization have been found to be conserved in monitor lizards (Varanidae) [18,68]. Conservation of several homologous syntenic regions has been found to be retained within different groups of fishes [69,70] and birds [71,72] as well. Comparative painting has also revealed chromosome homologies between bird groups [73] and also between vertebrate groups as observed between turtle sex chromosomes and amphibian autosomes [74]. The data presented in agamids show that this group has quite conserved karyotypes as well, and many rearrangements can be putatively dated a long time ago (Figure 3). The broad distribution of our *Pogona vitticeps* derived BAC sequences among agamids indicate that there has been conservation of chromosome segments across agamid lineages. The BAC sequence contained in the BAC Pv150_H19 is largely conserved in microchromosomes across the agamid phylogeny, while the other (BAC Pv03_L07) in macrochromosomes appears to have been only translocated to microchromosomes in the ancestor of the studied members of the subfamily Amphibolurinae. It was then likely later duplicated to a microchromosome containing the BAC sequence Pv150_H19, while the original microchromosome copy of the BAC Pv03_L07 has been lost in the ancestor of *P. vitticeps* (Figure 3). Since nearly half of the BAC Pv03_L07 consists of mobile elements, another explanation of the co-occurrence of this BAC signal in the microchromosomes could be as a result of the propagation of these mobile elements. The co-occurrence of both sequences in the ZW sex microchromosomes in *P. vitticeps* is thus likely a result of a rather complex history of rearrangements [59]. Future investigations that include more agamid lizards will better test the proposition that the reconstruction of events suggested here was important for the establishment of cytogenetically distinguishable sex chromosomes in *P. vitticeps* and its relatives.

## Figures and Tables

**Table 1:** Results of the bacterial artificial chromosome (BAC) clone fluorescence in situ hybridization (FISH) experiments. SDM = sex determination mechanism, 2n = diploid chromosome number, M + m = number of macrochromosomes and microchromosomes, GSD = genotypic sex determination, TSD = temperature-dependent sex determination, UNK = unknown, OP = unisexuality with obligatory parthenogenesis, qtel—telomeric region of the large arm of a macrochromosome.

Taxon	SDM	2n	M + m	Sex	Mapping	
					Pv03_L07	Pv150_H19
**Family: Agamidae**						
**Subfamily Amphibolurinae**						
*Pogona vitticeps*	GSD—ZW	32	12 + 20	1 F	2*qtel* + ZW micro sex chromosome	ZW micro sex chromosome
*Tympanocryptis lineata*	UNK	32	12 + 20	1 M, 1 F	2*qtel* + 1 pair of micros	1 pair of micros
*Rankinia diemensis*	UNK	32	12 + 20	1 M, 1 F	2*qtel* + 2 pairs of micros	1 pair of micros
**Subfamily Agaminae**						
*Agama picticauda*	TSD	44	20 + 24	1 M, 1 F	1*qtel*	1 pair of micros
*Phrynocephalus* cf. *guttatus*	UNK	46	22 + 24	1 M, 1 F	1*qtel*	No hybridization
**Subfamily Draconinae**						
*Calotes versicolor*	TSD	34	12 + 22	1 M, 1 F	2*qtel*	1 pair of micros
*Bronchocela cristatella*	UNK	34	14 + 20	1 F	5*qtel*	No hybridization
**Subfamily Hydrosaurinae**						
*Hydrosaurus* sp.	UNK	36	12 + 24	1 UNK	No hybridization	1 pair of micros
*Hydrosaurus weberi*	UNK	36	12 + 24	1 M	2*qtel*	No hybridization
**Subfamily Leiolepidinae**						
*Leiolepis reevesii rubritaeniata*	UNK	36	12 + 24	1 F	2*qtel*	1 pair of micros
*Leiolepis* cf. *ngovantrii*	OP	36	12 + 24	1 F	2*qtel*	1 pair of micros
**Subfamily Uromastycinae**						
*Saara loricata*	UNK	36	12 + 24	1 M, 1 F	2*qtel*	No hybridization
**Family: Chamaeleonidae**						
*Chamaeleo calyptratus*	XY	24	12 + 12	1 M, 1 F	No hybridization	No hybridization
*Trioceros johnstoni*	UNK	36	14 + 22	1 M, 1 F	No hybridization	No hybridization
